# Dysmenorrhea among high-school students and its associated factors in Kuwait

**DOI:** 10.1186/s12887-019-1442-6

**Published:** 2019-03-18

**Authors:** Sharefah Al-Matouq, Hessah Al-Mutairi, Ohood Al-Mutairi, Fatima Abdulaziz, Dana Al-Basri, Mona Al-Enzi, Abdullah Al-Taiar

**Affiliations:** Department of Community Medicine and Behavioural Sciences, Faculty of Medicine, Kuwait University, Box: 24923, 13110 Safat, Kuwait

**Keywords:** Dysmenorrhea, School girls, Kuwait, Menstrual pain

## Abstract

**Background:**

Although dysmenorrhea is not a life-threatening condition, it can cause a substantial burden on individuals and communities. There is no data on the prevalence of dysmenorrhea in Kuwait. This study aimed to estimate the prevalence of dysmenorrhea among female public high-school students in Kuwait and investigate factors associated with dysmenorrhea.

**Methods:**

A cross-sectional study using multistage cluster sampling with probability proportional to size method was conducted on 763 twelfth grade female public high-school students (aged 16–21 years). We used face-to-face interview with a structured questionnaire to collect data on dysmenorrhea and presumed risk factors. Weight and height of the students were measured using appropriate weight and height scales in a standardized manner. The association between dysmenorrhea and potential risk factors was assessed using multiple logistic regression.

**Results:**

The one-year prevalence of dysmenorrhea was found to be 85.6% (95%CI: 83.1–88.1%). Of the participants with dysmenorrhea, 26% visited a public or a private clinic for their pain and 4.1% were hospitalized for their menstrual pain. Furthermore, 58.2% of students with dysmenorrhea missed at least one school day and 13.9% missed at least one exam. Age of menarche (*p*-value = 0.005), regularity and flow of the menstrual period (p-value = 0.025, p-value = 0.009; respectively), and drinking coffee (p-value = 0.004) were significantly associated with dysmenorrhea in multivariable analysis.

**Conclusion:**

Dysmenorrhea seems to be highly prevalent among female high-school students in Kuwait, resembling that of high-income countries. Because of the scale of the problem, utilizing school nurses to reassure and manage students with primary dysmenorrhea and referring suspected cases of secondary dysmenorrhea is recommended.

## Background

Menstrual period is a cyclic physiological phenomenon; in which several problems can arise including irregular cycles, excessive bleeding, and dysmenorrhea. Dysmenorrhea is commonly described as a severe, painful, cramping sensation in the lower abdomen that is often associated with other symptoms, such as sweating, headaches, nausea, vomiting, and diarrhea [1]. These symptoms can occur during or a few days before menstruation. Dysmenorrhea can be primary, which is defined as pain without any clear pathological gynecological origin. Hypothesized pathways include endometrial release of large and imbalanced amounts of prostanoids and possibly eicosanoids during the menstrual cycle, which causes the uterus to contract frequently and dysrhythmically ultimately causing pain during menstruation [2]. On the other hand, secondary dysmenorrhea is caused by obvious underlying pelvic pathology and could occur years after menarche. It is associated with a variety of conditions including endometriosis, pelvic inflammatory disease, ovarian cysts, adenomyosis, and uterine myomas [3].

Although primary dysmenorrhea is not a life-threatening condition, it can cause a substantial burden on the quality of life of women or female adolescents [4–6]. A literature review of previous studies showed that dysmenorrhea negatively impacts the quality of life of affected women including their relationships with family members and friends, school or work performance in addition to social and recreational activities [7]. It has been also reported that women with dysmenorrhea tend to have higher sensitivity to pain in general even at the time when they have no menstrual pain [7]. Furthermore, dysmenorrhea is deemed to have significant economic consequences [8]. In the United States, the economic burden of dysmenorrhea has been estimated to be 600 million work hours or 2 billion dollars [9]. In Japan, it has been reported that the total healthcare cost for patients with primary dysmenorrhea is 2.2 times higher than the healthcare cost for females with no dysmenorrhea after adjusting for baseline characteristics [10].

There is large variation in the reported prevalence of primary dysmenorrhea between different countries and sometimes within the same country, which could be due to the use of different definitions of the condition [7]. Literature reviews of previous studies [5, 7] suggested that the prevalence of primary dysmenorrhea ranged from 34% or 45 to 95% among menstruating women. Dysmenorrhea tends to affect adolescent females more frequently than older women; therefore, results from studies reporting prevalence among adolescent girls cannot be generalized to older women [11]. As mentioned above, the lack of a uniform operational definition for dysmenorrhea to be used in epidemiological studies could be the underlying reason for the differences in the prevalence of primary dysmenorrhea between different settings.

Epidemiological studies have identified a number of factors associated with primary dysmenorrhea but the association between dysmenorrhea and many of these risk factors is still uncertain. In a review article, Ju et al. reported smoking, high body mass index (BMI), early age of menarche, longer and heavier menstrual flow, and family history of dysmenorrhea as predisposing factors for dysmenorrhea [12]. Although dysmenorrhea is deemed to be a sensitive issue in the Arab states in the Gulf region and Middle East, there have been some studies that explored the prevalence of dysmenorrhea [13–19] but none of these studies were in Kuwait. This study aimed to estimate the prevalence of dysmenorrhea among high-school female students and investigate factors associated with dysmenorrhea.

## Methods

### Study population and study participants

The total population of Kuwait is 4.2 million with 24.8% of the population below 20 years of age. The literacy in females aged between 15 to 24 years in Kuwait is 98.7% [20]. There are 77 female public high-schools in Kuwait with 40,095 students typically between the age 14 and 19 years. The study population was female students at the twelfth grade (typically aged 16–19) from public high-schools from all governorates in Kuwait. It was thought that this group would be easily accessible and would allow for a valid inference to be made on the prevalence of dysmenorrhea and its associated factors. The inclusion criteria were female students who attended their schools on the day of data collection and were willing to participate in the study.

### Study design and sampling methods

A cross-sectional study was conducted on a representative sample of female high-school students at twelfth grade (aged 16–21 years) that were selected using probability proportional to size sampling after stratification by governorate. Using the list of all female public high-schools (with the number of students in each school), multistage random cluster sampling was used to select the participants at public high-schools in all governorates of Kuwait. The relative size of each governorate, which was judged by the number of female students in high-schools, was taken into account to set the number of participants required.

### Data collection

Data on dysmenorrhea were gathered by face-to-face interviews conducted by six female senior medical students. The interview was based on a structured questionnaire that was developed after extensive review of the literature. The questionnaire comprised four major parts, which in addition to socio-demographic factors focused on the presence of dysmenorrhea, its associated symptoms (i.e. fatigue, headaches, breast tenderness … etc.), and impact on academic life. The presence of dysmenorrhea was assessed by asking the students if they had pain during their menstrual period in the past year. Participants who answered “yes, always”, “yes, often”, or “yes, sometimes” were considered to have dysmenorrhea; while those who answered “yes, rarely”, or “never” were considered to have no dysmenorrhea. A similar approach was used in a previous study in Canada [21]. The questionnaire was developed in English and was translated into Arabic and then back-translated to English by an independent person who was not part of this study. The original English questionnaire and the back-translated were then compared. The final Arabic version of the questionnaire was pretested on 20 students from the same age group, who were not included in the study.

The menstrual pain was assessed using a horizontal visual analog scale (VAS) with a 100-mm line; one end of the line represents “no pain” and the other end represents “worst possible pain”. The participants were asked to rate the degree of their pain by making a mark on the line. The data collectors then measured the answers marked by the students with a ruler. The scores received from the scale were classified into mild (> 5 to ≤44 mm), moderate (> 44 to ≤74 mm), and severe (> 74 mm) [22]. The question regarding the location of the menstrual pain was illustrated using photo cards and the students were allowed to choose more than one site. Data on regularity of menstrual period were gauged using the question “Do you describe your period as being regular or irregular?” with the answers “regular or irregular”. Data on the management of pain were gathered using the question “What do you do to relieve your pain?” followed by a list of options (bed rest, heating pad, medication prescribed by a doctor, medication taken by myself, tea/herbs and others) with the student allowed to selected more than one option. Similarly data on symptoms were collected by the question “Do you experience any of the following symptoms during your period?” followed by a list of symptoms with the student allowed to select more than one option.

We collected data on socio-demographic factors in addition to hypothesized risk factors for dysmenorrhea such as characteristics of the menstrual period, smoking, second-hand smoking, consumption of specific dietary items and drinks. We also collected data on presence of any disease condition that was diagnosed by a medical doctors. Furthermore, we collected data on physical activity using a self-administered questionnaire, which has already been translated to Arabic and validated in our setting using accelerometers (Spearman correlation 0.92; *p* < 0.001 for total steps count) (not published). This questionnaire contained 14 questions, asking about different physical activities that participants did in the previous 7 days with their frequency and duration. We measured height using a portable stable stadiometer (SECATMR) to the nearest 0.1 cm, and weight using digital weight scale (BeurerR) to the nearest 0.1 kg.

### Data analysis

Data were entered and analyzed using Statistical Package for Social Sciences (SPSS). Using the measured weight and height, BMI was calculated (weight (Kg)/height(m^2^)) and categorized into underweight, normal weight, overweight and obese for students below the age of 18 years according to WHO’s growth charts, and for those ≥18 years as per WHO’s classification (underweight< 18, normal 18.5 to 24.9, overweight 25 to 29.9, obese ≥30). The 95% CI for the one-year prevalence of dysmenorrhea was calculated using the exact binominal distribution. We used Chi-square test to investigate the association between dysmenorrhea and categorical variables. The adjusted association between dysmenorrhea and multiple factors was assessed using multiple unconditional logistic regression. The binary outcome in this analysis was created by categorizing participants who reported always, often, or sometimes having pain with their menstrual period during the past year in one group (dysmenorrhea group), while those who reported having pain with their menstrual period rarely or never in another group (no dysmenorrhea group).

### Ethical approval

The study was approved by The Health Sciences Center Ethics Committee at Kuwait University (Ref: 3660–16/10/2017). We also obtained the permission from The Ministry of Education in Kuwait. Each participant completed a written informed consent, which outlined the objectives of the study before the interview was initiated.

## Result

Of 787 students who were approached, 766 (97.3%) agreed to participate. Three participants have not reached menarche, and therefore, were excluded from the study. Thus, the analysis below comprised 763 participants. Table 1 shows the socio-demographic characteristics of the study participants. The mean (SD) age of the participants was 17.4 (0.7) years. Table 2 shows the description of the menstrual period of 763 female high-school students. The mean (SD) age of menarche was 12.1 (1.3) years; and about half of the study group described their menstrual period as irregular; while 56.1% reported having their period lasting between 6 and 8 days.

**Table 1:** Socio-demographic characteristics of 763 female public high-school students in Kuwait, 2017

	Number	(Percent)
Age in years, mean (SD)	17.4 (0.7)	
Nationality		
Kuwaiti	655	(85.8)
Non-Kuwaiti	108	(14.2)
Father’s education^a^		
Intermediate/below	98	(12.9)
Secondary (high school)	230	(30.2)
Diploma	64	(8.4)
University and above	369	(48.5)
Mother’s education		
Intermediate/below	132	(17.3)
Secondary (high school)	196	(25.7)
Diploma	76	(10.0)
University and above	359	(47.0)
Father’s income per month^b^		
Less than 500 KD	25	(3.3)
500–1000 KD	93	(12.2)
1001–1500 KD	140	(18.4)
1501–2000 KD	99	(13.0)
More than 2000 KD	77	(10.1)
Do not wish to tell	328	(43.0)
Mother’s income per month		
Less than 500 KD	102	(13.4)
500–1000 KD	174	(22.8)
1001–1500 KD	88	(11.5)
1501–2000 KD	73	(9.6)
More than 2000 KD	33	(4.3)
Do not wish to tell	293	(38.4)
Currently lives with:		
Both parents	671	(87.9)
Mother without the father	73	(9.6)
Father without the mother	15	(2.0)
Other relatives	4	(0.5)
Number of sisters		
≤ 2	448	(58.7)
3–4	214	(28.1)
≥ 5	101	(13.2)
Number of brothers		
≤ 2	421	(55.2)
3–4	272	(35.6)
≥ 5	70	(9.2)

^a^ Missing for two participants; ^b^ Missing for one participant

**Table 2:** Description of menstrual period of 763 female high-school students in Kuwait, 2017

	Number	Percent
Age of menarche, mean (SD) years	12.1 (1.3)	
Regularity of menstrual period^a^		
Regular	367	(48.1)
Irregular	396	(51.9)
Duration of menstrual period (days)		
≤ 3	31	(4.1)
4–5	268	(35.1)
6–8	428	(56.1)
> 8	36	(4.7)
Number of pads changed in the first three days of period		
≤ 3	240	(31.5)
4–5	351	(46.0)
≥ 6	172	(22.5)
Flow of menstrual period (as described by the participants)^b^		
Mild	59	(7.7)
Moderate	467	(61.3)
Heavy	115	(15.1)
Heavy with clots	121	(15.9)

^a^ As described by the study participants. ^b^ Missing for one participant

### Prevalence of dysmenorrhea

The prevalence of dysmenorrhea in addition to the description of the pain intensity and its duration are shown in Table 3. Of 763 participants, 653 (85.6%; 95%CI: 83.1–88.1%) had dysmenorrhea according to the definition used in our study (those who reported pain with their menstrual period always, often, or sometimes during last year). This was 560 (85.5%) and 93 (86.1%) among Kuwaitis and non-Kuwaitis, respectively (*p*-value = 0.866). There was no significant difference in the prevalence of dysmenorrhea between different governorates (p-value = 0.137). If we include those who reported having the menstrual pain rarely, the prevalence becomes 702 (92.0%; 95%CI: 90.0–93.9%). In other words, only around 8.0% reported that they have never had pain with their menstrual period.

**Table 3:** Description of dysmenorrhea and the characteristics of pain experienced by 653 female students from Kuwait public high-schools, 2017

Questions	Number	%	n(%)
During the last year:			
Did you have pain with your menstrual period? (*N* = 763)			
Yes, always	353	(46.2)	653 (85.6)
Yes, often	131	(17.1)	
Yes, sometimes	169	(22.1)	
Yes, rarely	50	(6.5)	110 (14.4)
Never	60	(7.9)	
How long does the pain usually last? (N = 653)			
Less than one day	102	(15.6)	
1–2 days	364	(55.7)	
3–4 days	163	(25.0)	
More than 4 days	24	(3.7)	
Where do you experience the pain?^a^ (*N* = 653)			
Lower abdomen	640	(98.0)	
Back	455	(69.7)	
Thighs	189	(28.9)	
Legs	84	(12.9)	
Have you visited a public/private clinic for this pain? (yes)(N = 653)	170	(26.0)	
Have you been hospitalized for the pain? (yes)(N = 653)	27	(4.1)	
Pain severity (N = 653)			
Mild (5–44)	119	(18.2)	
Moderate (45–74)	333	(51.0)	
Severe (75–100)	201	(30.8)	
Have you ever missed a school day because of your menstrual pain? (yes)(N = 653)	380	(58.2)	
Have you ever missed an exam because of the menstrual pain? (yes)(N = 653)	91	(13.9)	
Does your pain severity change throughout the year depending on the weather? (yes))N = 653)	305	(46.7)	
Is it worse in cold or hot seasons? (N = 653)			
Hot seasons	39	(6.0)	
Cold seasons	266	(40.7)	

^a^ Participants can select more than one option

**Fig. 1:**
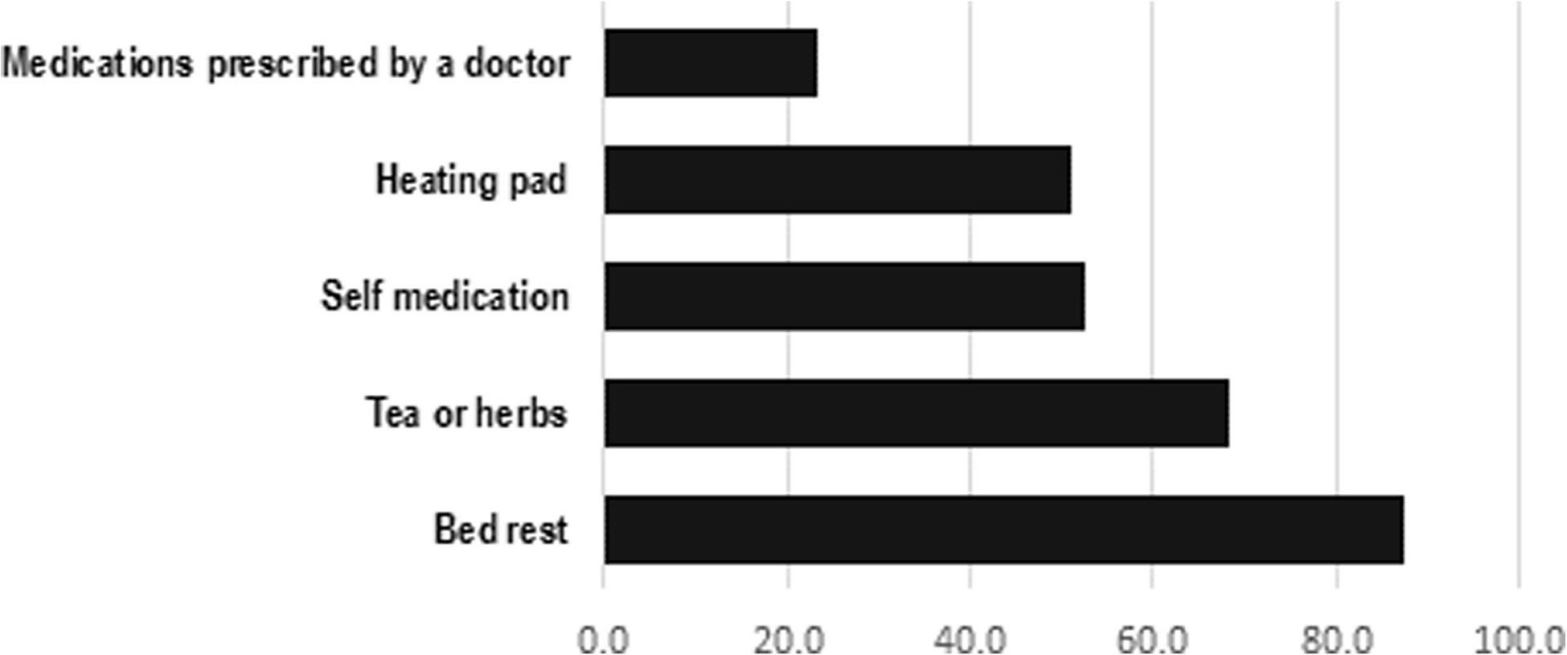
Management of menstrual pain by 653 female public high-school students with dysmenorrhea, Kuwait 2017

**Fig. 2:**
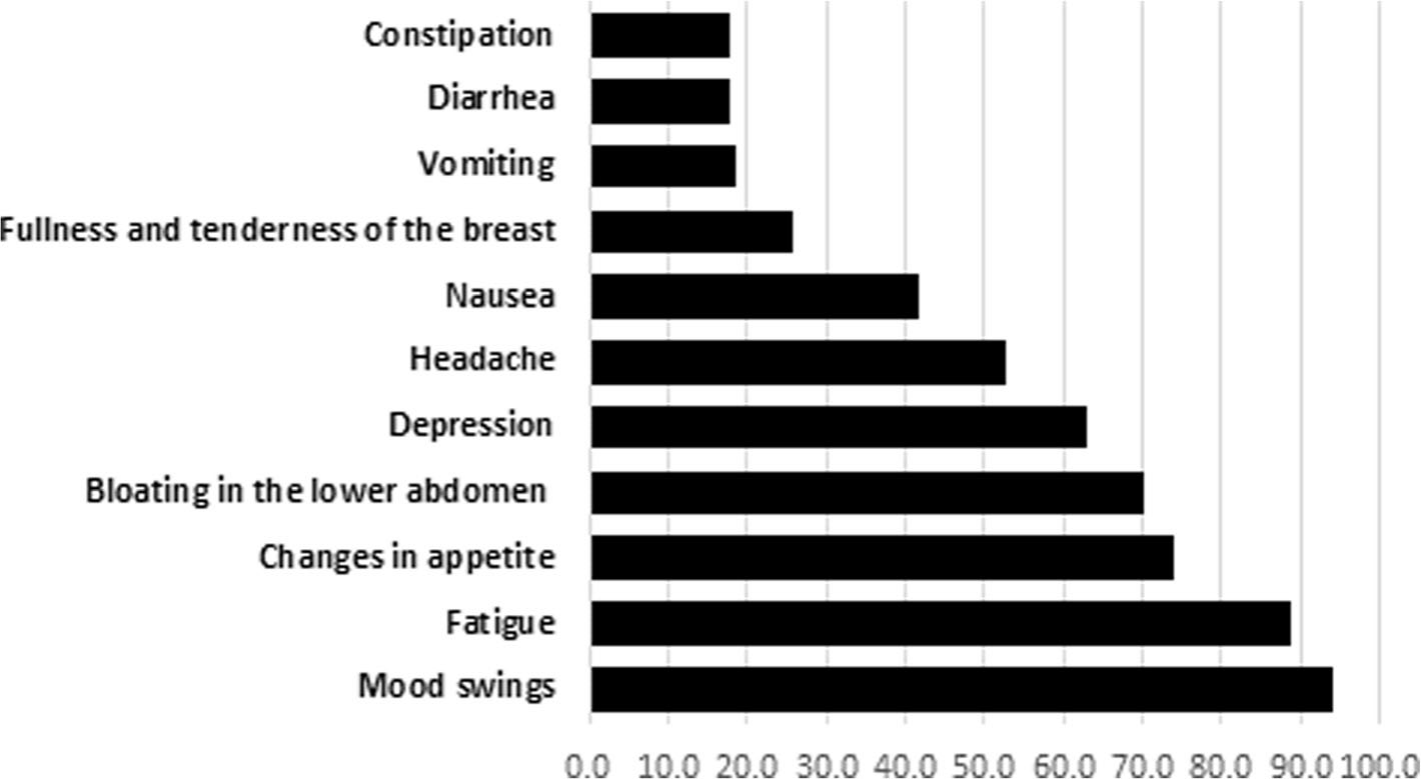
Reported symptoms of dysmenorrhea during the menstrual period by 763 female public high-school students, Kuwait 2017

**Fig. 3:**
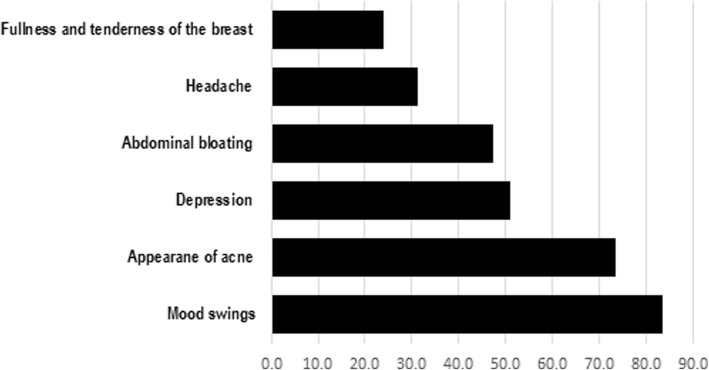
Reported symptoms of dysmenorrhea before the menstrual period by 763 female public high-school students, Kuwait 2017

More than half of the participants with dysmenorrhea had pain for 1 to 2 days; and the most common site for pain was the lower abdomen. It is worth noting that 26% of the participants with dysmenorrhea (170 out of 653) have visited a public or private clinic because of their pain. Out of students with dysmenorrhea (*N* = 653), 27 (4.1%) were hospitalized for the management of their menstrual pain. Methods the participants used to relieve their pain are shown in Fig. 1, while the most common symptoms during and before the menstrual period are shown in Figs. 2 and 3. More than half of the participants with dysmenorrhea have missed at least one school day during the last academic year. Also, 91 (13.9%) of the participants with dysmenorrhea missed at least one school exam.

### Risk factors for dysmenorrhea

Table 4 shows the association between dysmenorrhea and presumed risk factors in univariable analysis. Factors that showed significant association with dysmenorrhea in univariable analysis were age of menarche, the regularity of the menstrual period as reported by the participants, the flow of the menstrual period and the frequency of drinking coffee. Table 5 shows the association between dysmenorrhea and risk factors in multivariable logistic regression analysis. The age of menarche was found to be significantly associated with dysmenorrhea; adjusted odds ratio 0.80 (95%CI: 0.69–0.93), (*p*-value = 0.005). Similarly, having irregular menstrual period, as described by the participants, was negatively associated with dysmenorrhea; adjusted odds ratio 0.59 (95%CI: 0.38–0.91), (p-value = 0.018). The flow of menstrual period as reported by the participants was also positively associated with dysmenorrhea (p-value = 0.006). Furthermore, drinking coffee four or more times per week was positively associated with dysmenorrhea, adjusted odds ratio 2.19 (95%CI: 1.39–3.44), (p-value = 0.001). We also repeated the analysis after recoding those who rarely had menstrual pain as dysmenorrhea (i.e. never had menstrual pain vs. rarely, sometimes, often, and always had menstrual pain). In multivariable analysis, only age of menarche, regularity and flow of the menstrual period and weekly drinking of coffee were significantly associated with dysmenorrhea. These findings are identical to the results from the previous analysis.

**Table 4:** Association between dysmenorrhea and potential risk factors among 763 female high-school students in Kuwait in univariable analysis

	OR^a^	[95% CI]	*p*-value^b^
Variables			
Nationality			
Non-Kuwaiti	1.00	[Reference]	0.866
Kuwaiti	0.95	[0.53–1.71]	
Father’s education			
Intermediate school or below^c^	0.84	[0.46–1.55]	0.830
Secondary (high-school)	1.05	[0.65–1.70]	
Diploma	0.79	[0.39–1.61]	
University and above	1.00	[Reference]	
Mother’s education			
Intermediate school or below^c^	0.89	[0.51–1.55]	0.419
Secondary (high-school)	1.34	[0.78–2.28]	
Diploma	0.75	[0.39–1.44]	
University and above	1.00	[Reference]	
Currently lives			
With mother	1.00	[Reference]	0.637
Without the mother	1.43	[0.32–6.27]	
Number of sisters			
≤ 2	1.00	[Reference]	0.317
3–4	0.85	[0.54–1.33]	
≥ 5	1.52	[0.75–3.06]	
Number of brothers			
≤ 2	1.00	[Reference]	0.183
3–4	1.32	[0.84–2.08]	
≥ 5	0.70	[0.37–1.34]	
Passive smoking at household			
Yes	0.89	[0.59–1.33]	0.565
No	1.00	[Reference]	
Sleep hours per night during week days			
< 6 h	1.64	[0.95–2.82]	0.201
6–7 h	1.18	[0.74–1.89]	
> 7 h	1.00	[Reference]	
Sleep hours per night during weekends			
< 8 h	1.25	[0.74–2.133]	0.647
8–10 h	1.19	[0.75–1.92]	
> 10 h	1.00	[Reference]	
Presence of disease condition diagnosed by doctor			
No	1.00	[Reference]	1.21
Yes	1.21	[0.75–1.95]	
Taking supplements			
No	1.00	[Reference]	0.269
Yes	1.29	[0.82–2.02]	
Taking medication			
No	1.00	[Reference]	0.077
Yes	2.06	[0.92–4.58]	
History of surgeries			
No	1.00	[Reference]	0.143
Yes	1.49	0.87–2.55	
Age of menarche	0.81	[0.70–0.94]	0.005
Regularity of menstrual period^d^			
Regular	1.00	[Reference]	0.025
Irregular	0.62	[0.41–0.94]	
Duration of menstrual period			
≤ 3	1.00	[Reference]	0.081
4–5	2.48	[1.06–5.78]	
6–8	2.77	[1.21–6.33]	
> 8	1.69	[0.55–5.26]	
Number of pads changed in the first three days of the period			
4–5	1.00	[Reference]	0.323
≤ 3	0.624	[0.39–0.99]	
≥ 6	0.743	[0.44–1.26]	
Flow of menstrual period^d^			
Mild	0.38	[0.17–0.86]	0.009
Moderate	0.70	[0.38–1.30]	
Heavy	1.75	[0.70–4.34]	
Heavy with clots	1.00	[Reference]	
Family history of menstrual pain			
No	1.00	[Reference]	0.298
Yes	1.29	[0.79–2.1]	
Diet description by the participant			
I eat meat, fish, or both	1.00	[Reference]	0.398
I don’t eat meat or fish/I am vegetarian and eat eggs/I am vegetarian and don’t eat eggs	1.57	[0.55–4.51]	
Weekly consumption of fast food			
≤ 2 days	1.00	[Reference]	0.337
≥ 3 days	0.81	[0.52–1.25]	
Weekly fried food consumption			
≤ 2 days	1.00	[Reference]	0.344
≥ 3 days	1.22	[0.81–1.83]	
Energy drinks consumption			
No	1.00	[Reference]	0.592
Yes	0.88	[0.56–1.39]	
Weekly drinking of coffee			
≤ 3 times per week	1.00	[Reference]	0.004
≥ 4 times per week	1.9	[1.23–2.96]	
Weekly drinking of tea			
≤ 3 times per week	1.00	[Reference]	0.497
≥ 4 times per week	1.21	[0.7–2.07]	
Weekly drinking of green tea			
≤ 3 times per week	1.00	[Reference]	0.547
≥ 4 times per week	1.28	[0.57–2.9]	
Weekly drinking of carbonated drinks			
≤ 3 times per week	1.00	[Reference]	0.371
≥ 4 times per week	0.83	[0.54–1.25]	
BMI^e^			
Normal	1.00	[Reference]	0.212
Overweight	0.82	[0.49–1.35]	
Obese	0.59	[0.37–0.96]	
Physical activity in the previous week^f^			
< 3.67 h	1.00	[Reference]	0.983
3.67–9.50 h	0.95	[0.57–1.58]	
> 9.50 h	0.97	[0.58–1.61]	

^a^ Crude unadjusted odds ratio; ^b^ *P*-values were generated using Chi-square test; ^c^ Intermediate school or below group included non-educated and primary level education; ^d^ as reported by the participants; ^e^ Weight and height were measured and for those who are below 18 years old, z-score was calculated using WHO’s growth chart, otherwise WHO’s BMI classification was used; ^f^ It includes only 737 participants

**Table 5:** Association between dysmenorrhea and potential risk factors among 763 female high-school students in Kuwait in multivariable analysis

Variables	OR^a^	[95% CI]	*p*-value^b^
Age of menarche	0.80	[0.69–0.93]	0.005
Regularity of menstrual period			
Regular	1.00	[Reference]	0.018
Irregular	0.59	[0.38–0.91]	
Flow of menstrual period^c^			
Mild	0.44	[0.19–1.01]	0.006
Moderate	0.78	[0.41–1.47]	
Heavy	2.10	[0.83–5.31]	
Heavy with clots	1.00	[Reference]	
Weekly drinking of coffee			
≤ 3 times per week	1.00	[Reference]	0.001
≥ 4 times per week	2.19	[1.39–3.44]	

^a^ Adjusted odds ratio; ^b^ p-values were generated using likelihood ratio test; ^c^ as reported by the participants

## Discussion

This study aimed to estimate the prevalence of dysmenorrhea among female public high-school students in Kuwait and to explore the relationship between dysmenorrhea and several presumed risk factors. There is a paucity of data on dysmenorrhea and its associated factors in Kuwait. We have demonstrated that the majority of female students in Kuwait had dysmenorrhea and that a large number had sought medical treatment from private or public healthcare services.

The one-year prevalence of dysmenorrhea was found to be 85.6%. Because pain is a highly subjective symptom and therefore difficult to quantify, there is a lack of consensus on an operational definition of dysmenorrhea in epidemiological studies. As a result, it is difficult to compare our findings with that of other studies at a regional or an international level. Unfortunately, in some studies, the definition of dysmenorrhea was not clearly stated [23, 24]; while in other studies dysmenorrhea was defined as pain with menstrual period without further specification of intensity and/or frequency [25, 26]. Developing a consensus on a standard definition of dysmenorrhea is an important step to study the geographical distribution of dysmenorrhea and its trends over time.

Overall, the one-year prevalence was very high in our setting similar to that reported in other studies in the region such as Saudi Arabia (60.9% among female medical students) [13], Oman (94% among high-school students) [14], and Iran (98.4% among female medical students) [27]. Our findings are also consistent with that reported from high-income countries such as Canada (60% in girls aged 18 and above) [21] and Australia (88% among females aged 16 to 25 years old) [28].

Dysmenorrhea can be a major cause for school absenteeism and missing exams. In Saudi Arabia, of the university students with dysmenorrhea, 28.3% had absenteeism [13], while in Turkey 32% of female high-school students reported school absenteeism due to dysmenorrhea [29]. In our study, 58.2% of students with dysmenorrhea missed at least one school day during the last academic year. Also, 13.9% of the participants with dysmenorrhea missed at least one exam in the past academic year. The difference between our findings and other studies could be due to an increased tendency to report dysmenorrhea as a reason for school absenteeism among female high-school students compared to students in other countries. Approximately, 26% of the participants with dysmenorrhea visited public or private clinic because of their menstrual pain, which is different from that reported amongst Hispanic adolescents in Texas (only 14% of school girls with dysmenorrhea had sought physician advice) [30] or Egyptian girls (the majority of students did not seek medical advice for dysmenorrhea) [18]. The easier access to healthcare services in Kuwait could explain the higher proportion of adolescents seeking medical care for dysmenorrhea compared to other settings.

Significant association between early age of menarche and dysmenorrhea was found in univariable and multivariable analysis (Tables 4 and 5), which could be due to the fact that early menarche reflects longer exposure to uterine prostaglandins that plays a major role in dysmenorrhea through increasing uterine contractility resulting in pain [31]. Another reason could be that dysmenorrhea typically occurs with ovulatory cycles, which are not established immediately after menarche [32]. Therefore, later onset of menarche means that females have unovulatory cycles and are less likely to report pain; although this does not mean that they will not experience dysmenorrhea later on in their life.

Surprisingly, irregular menstrual period (as described by the participants) was found to be negatively associated with dysmenorrhea. We found no evidence in literature suggesting that having irregular periods is protective against dysmenorrhea. In fact, several studies have demonstrated a positive relationship between having irregular periods and dysmenorrhea [11, 33]; while other studies reported no association between dysmenorrhea and regularity of the menstrual period [14, 34]. In our study, it is possible that females with dysmenorrhea were more likely to report their menstrual period as being regular since they anticipate their period (hence their pain) each month. We also found that the flow of the menstrual period (the amount of blood lost during a menstrual period as reported by the participants) was significantly associated with dysmenorrhea, which is consistent with several studies [13, 35]. Both the flow of menstruation and dysmenorrhea are thought to be determined by prostaglandins. In case of increased blood flow, prostaglandins can disturb the homeostatic mechanism of the endometrium; hence, increasing the blood flow. Moreover, platelets aggregation and/or various coagulation factors are affected by prostaglandins leading to the increase of menstrual blood flow [36, 37]. It is also possible that the link between the flow of the menstrual period and dysmenorrhea is not genuine and that females who experienced pain were more likely to report their period as being heavy (recall bias).

Drinking coffee was the only modifiable risk factor that showed an association with dysmenorrhea in our analysis. Drinking coffee four or more times per week was positively associated with dysmenorrhea in univariable and multivariable analysis (Tables 4 and 5). Results from studies that assessed the association between coffee drinking and dysmenorrhea are controversial. Some studies reported positive association between coffee drinking and dysmenorrhea [38, 39]; while others showed no association between dysmenorrhea and daily caffeine intake [34, 40–42]. Caffeine, which is the main ingredient of coffee, is an adenosine analogue that inhibits adenosine (a potent vasodilator) receptors [43]. Blocking these receptors causes vasoconstriction that will decrease the blood flow to the uterus causing further increase in the degree of menstrual pain [44]. In our study, drinking coffee was common (more than 37% of the study group consumed coffee 6 or more times per week), but consumption of other caffeine drinks such as energy drinks was too low, and thus, we were unable to look for the association between other caffeinated drinks and dysmenorrhea.

Similar to other studies [45, 46], no significant association was found between physical activity and dysmenorrhea. Measuring physical activity among young adults is difficult and may result in a substantial non-differential misclassification, which can explain the lack of association between physical activity and dysmenorrhea in our study and other studies [45, 46]. In addition to the studies that showed no association between dysmenorrhea and physical activity, a study in Japan reported that physical activity is inversely associated with dysmenorrhea [47]. However, with the cross-sectional design, the relationship could be explained by the reverse-causality (girls with dysmenorrhea and heavy blood flow may refrain from exercise and other physical activities). Prospective studies are better to address this question and other questions related to the risk factors of dysmenorrhea.

This is the first study to explore the prevalence of dysmenorrhea and its related factors in Kuwait. We used a nationally representative sample; and only few students refused to participate. There are several limitations in the study, including the inherent weakness of the cross-sectional design, which does not allow for causal inferences. As an example, it is not clear if physical inactivity predisposes to dysmenorrhea or dysmenorrhea itself hinders physical activity (in our study we found no association between physical activity and dysmenorrhea). Finally, it is possible that students with severe menstrual pain (dysmenorrhea) were absent during the data collection, which would underestimate the prevalence of dysmenorrhea. However, the high prevalence we reported does not suggest that we missed a large number of students with dysmenorrhea.

## Conclusion

Dysmenorrhea seems to be highly prevalent among female high-school students in Kuwait, resembling that of high-income countries. A substantial number of those with dysmenorrhea visited private or public healthcare services and also missed school days and school exams. Univariable and multivariable analysis have shown that age of menarche, regularity and flow of the menstrual period, and coffee drinking are significant independent predictors for dysmenorrhea. Although dysmenorrhea is not a life-threatening health condition, our data suggest dysmenorrhea as a major public health problem among female high-school students causing social burden on families and students alike in addition to affecting school attendance. In Kuwait, each school has a clinic with a nurse, which can be utilized to manage and reassure girls with dysmenorrhea. Training school nurses on management of primary dysmenorrhea and referral of suspected cases of secondary dysmenorrhea seems to be logical in the light of the size of the problem. Currently, the literature shows contradictory results with respect to the factors associated with dysmenorrhea which could be due to the lack of a uniform operational definition of dysmenorrhea in epidemiological studies. The only modifiable risk factor we found was drinking coffee. Although this is biologically plausible, the studies are inconclusive about this issue. Avoidance of heavy caffeine consumption is recommended for several other reasons and might be also useful for reducing dysmenorrhea.

## Abbreviations

95%CI: 95% Confidence interval
BMI: Body mass index
SD: Standard deviation
SPSS: Statistical Package for Social Sciences
VAS: Visual analog scale
